# The Value of Electroacupuncture in the Treatment of Coronary Heart Disease: A Review of the Mechanisms and Clinical Studies of Electroacupuncture Therapy

**DOI:** 10.1155/crp/4684871

**Published:** 2025-07-01

**Authors:** Yuming Shao, Yang Li, Bing Wang, Chengjia Li, Huijun Chen

**Affiliations:** ^1^Heilongjiang University of Chinese Medicine, 24 Heping Road, Xiangfang District, Harbin 150040, China; ^2^First Affiliated Hospital, Heilongjiang University of Chinese Medicine, 26 Heping Road, Xiangfang District, Harbin 150040, China; ^3^Second Affiliated Hospital, Heilongjiang University of Chinese Medicine, 411 Guogeli Street, Nangang District, Harbin 150001, Heilongjiang, China

**Keywords:** coronary heart disease, electroacupuncture pretreatment, electroacupuncture therapy

## Abstract

Electroacupuncture (EA) therapy combines electrical stimulation with traditional acupuncture therapy and is widely used as a physical therapy in various fields. Coronary heart disease (CHD) is a prevalent cardiovascular condition. Applying EA in the treatment of CHD patients has proven to effectively enhance therapeutic outcomes and improve prognosis. This paper summarizes the potential mechanisms of EA in the treatment of CHD, its therapeutic effects on CHD patients, and analyzes the current bottlenecks in the application of EA therapy for CHD. Furthermore, it discusses potential future directions for EA in CHD management.

## 1. Introduction

Electroacupuncture (EA) therapy refers to a technique in which acupuncture needles are inserted into acupoints and connected to an EA apparatus. By applying microcurrent pulses similar to the body's bioelectricity, EA stimulates and regulates the meridian system to prevent and treat diseases [1]. The origins of EA therapy can be traced back to the late 19th and early 20th centuries [2]. With the global dissemination of Traditional Chinese Medicine (TCM), EA therapy has gradually gained acceptance among practitioners and patients worldwide. As a simple, safe, and effective treatment modality, EA has demonstrated significant potential in the prevention and management of diseases and their associated complications, with its efficacy validated in various clinical conditions. Coronary heart disease (CHD) is characterized by myocardial ischemia, hypoxia, or necrosis caused by coronary artery atherosclerosis (AS), leading to narrowing, spasm, or occlusion of the coronary arteries. Also known as ischemic heart disease, CHD has high morbidity and mortality rates, posing a substantial global health burden [3]. Myocardial infarction (MI) is a common clinical presentation of CHD. Early intervention in MI can restore blood flow to the ischemic myocardium and reduce mortality risk. However, the restoration of blood flow after a period of ischemia can result in further myocardial damage, including alterations in myocardial ultrastructure, function, metabolism, and electrophysiology, a phenomenon known as myocardial ischemia/reperfusion injury (MI/RI) [4]. MI/RI remains a major challenge in optimizing revascularization outcomes in CHD patients, particularly those with MI. Recent studies have demonstrated that EA pretreatment induces moderate stress responses, activating endogenous protective mechanisms akin to myocardial ischemic preconditioning. This has shown significant protective effects against myocardial injury caused by ischemia–reperfusion (I/R) [5]. This review aims to explore the mechanisms underlying the adjunctive use of EA in CHD treatment and its impact on patients, providing a reference for further application of EA therapy in CHD management.

## 2. Potential Mechanisms of EA in the Treatment of CHD

### 2.1. EA Reduces Inflammatory Responses

Inflammatory responses play a critical role in the pathological progression following acute MI (AMI). This process involves the generation of reactive oxygen species (ROS), complement cascade activation, and the recognition and combination of damage-associated molecular patterns (DAMPs) by pattern recognition receptors (PRRs) such as toll-like receptors (TLRs) and nucleotide-binding oligomerization domain (NOD)–like receptors (NLRs). However, excessive inflammatory responses can lead to adverse cardiac remodeling [6].

Nuclear factor-κB (NF-κB) is a key regulator of the inflammatory cascade, with the nuclear translocation of its p65 subunit being a critical step in pathway activation [7, 8]. Calcitonin gene–related peptide (CGRP), one of the most potent anti-inflammatory neuropeptides, is released upon the activation of transient receptor potential vanilloid 1 (TRPV1) [9]. The binding of CGRP to its receptor inhibits NF-κB activation, thereby mitigating local tissue inflammation [10]. Wu [11] observed that EA pretreatment at Neiguan (PC6) enhanced the expression of the TRPV1/CGRP signaling pathway in acute ischemic myocardial tissue, reduced NF-κB p65 expression, regulated inflammatory cytokine release, and decreased the extent of inflammatory cell infiltration. These effects improved cardiac function and reduced infarct size in AMI rats.

The phagocytosis and clearance of apoptotic cardiomyocytes by neutrophils can induce macrophage differentiation toward the M2 phenotype, promoting the secretion of anti-inflammatory and profibrotic cytokines, thereby facilitating the resolution of inflammation and the repair of damaged myocardial tissue [12]. Several studies have demonstrated that EA pretreatment regulates neutrophil infiltration and macrophage polarization, facilitating the transition of damaged myocardium from an acute proinflammatory phase to an anti-inflammatory reparative phase, thereby exerting cardioprotective effects [13–15].

TLR4 is highly expressed in the heart and plays a key role in initiating and regulating inflammatory responses. Activated TLR4 promotes the expression of proinflammatory factors such as interleukin-23 (IL-23), IL-17, and tumor necrosis factor-alpha (TNF-alpha) through the activation of NF-κB [16, 17]. Sun et al. [18] found that EA therapy reduced inflammatory cell infiltration and cardiomyocyte apoptosis in the myocardial tissue of MI rats. They suggested that EA's cardioprotective effects were primarily mediated by the inhibition of TLR4 expression in infarcted myocardial tissue, leading to the reduced release of IL-23 and IL-17, thereby mitigating inflammation and promoting the repair of damaged myocardial tissue. Similarly, Tang et al. [19] observed a reduction in myocardial infarct size and significantly decreased levels of TNF-alpha and IL-6 in the myocardial tissue of rats treated with EA pretreatment, indicating that EA pretreatment alleviates myocardial inflammatory injury by downregulating the expression of proinflammatory cytokines. Another study demonstrated that EA at Neiguan (PC6), Xinshu (BL15), and Geshu (BL17) reduced serum IL-23/IL-17 axis expression in mice, alleviating inflammation-induced vascular endothelial injury, decreasing macrophage and neutrophil recruitment, and achieving therapeutic effects in AS [20].

NOD-like receptor pyrin domain containing 3 (NLRP3) is a pattern recognition receptor that, upon activation, forms the NLRP3 inflammasome with caspase-1. A study confirmed that EA pretreatment at Neiguan (PC6) inhibits NLRP3 inflammasome activation, macrophage polarization toward the M1 phenotype, and neutrophil infiltration. These actions reduce inflammatory responses in ischemic myocardial tissue, decrease infarct size, and alleviate cardiac function damage, ultimately protecting ischemic myocardium [21].

### 2.2. EA Reduces Oxidative Stress (OS)

OS is a key factor in the occurrence of hypoxia and I/R-related cardiovascular diseases [22]. Broadly, OS refers to an imbalance between the production of ROS and the regulation of ROS metabolism or degradation by various antioxidant mechanisms, leading to an overproduction of free radicals, which in turn causes a series of harmful effects [23]. OS can cause highly reactive oxygen free radicals to react with unsaturated fatty acids on cell membranes, leading to lipid peroxidation [24]. Malondialdehyde (MDA), one of the final products of lipid peroxidation, is commonly used to measure the extent of oxidative damage [25]. Antioxidant enzymes, including superoxide dismutase (SOD), glutathione (GSH) peroxidase (GSH-Px), and catalase (CAT), can eliminate the excess accumulation of oxygen free radicals and reduce the detrimental effects of OS [26]. Tong et al. [27] found that EA therapy can lower serum MDA levels and increase SOD content in patients with angina, suggesting that EA therapy inhibits lipid peroxidation reactions triggered by oxygen free radicals, thereby alleviating oxidative damage to myocardial cells.

The nuclear factor erythroid 2–related factor 2 (Nrf2)–antioxidant response element (ARE) signaling pathway is one of the key pathways in cellular antioxidant stress. Nrf2, as an important transcription factor regulating OS in the body, when activated, promotes the expression of downstream antioxidant proteins, such as heme oxygenase-1 (HO-1), thus mitigating OS responses during MI/RI [28]. Shao et al. [29] found that after EA pretreatment at Jiaji (EX-B2) in rats, the expression of Nrf2 and HO-1 genes in myocardial tissue was significantly upregulated, suggesting that EA pretreatment may exert protective effects on the myocardium by activating the Nrf2–ARE signaling pathway.

Connexin 43 (Cx43), the primary protein in gap junctions, undergoes reduced expression and dephosphorylation, leading to the occurrence of arrhythmias during myocardial ischemia [30]. Some studies suggest that OS is involved in the process of Cx43 dephosphorylation [31]. Research has shown that EA not only regulates the release of antioxidant enzymes but also enhances Cx43 expression and improves its distribution, thereby protecting the myocardium of rats with chronic myocardial ischemia [32].

The NPPB gene encodes probrain natriuretic peptide, and abnormal expression of this gene has been confirmed to be associated with the occurrence of CHD [33]. Huang et al. [34] observed that EA pretreatment at Neiguan (PC6) significantly reduced the expression of the NPPB gene. This finding suggests that EA pretreatment can reduce OS-induced damage in myocardial tissue during I/R by regulating the expression of specific functional genes.

GSH is a key nonprotein thiol compound that resists OS and protects cells from damage caused by lipid peroxides and ROS [35]. There is a strong correlation between depression and CHD, and OS is one of the pathological mechanisms underlying the comorbidity of CHD and depression [36]. Studies have shown that EA therapy can increase GSH and CAT levels, reduce OS, and provide therapeutic effects for CHD complicated with depression [37, 38].

### 2.3. EA Regulates Autophagy Levels

Autophagy is a lysosome-mediated process for the degradation and recycling of unnecessary and dysfunctional proteins, organelles, and other cellular components [39]. During myocardial ischemia, autophagy plays a protective role in myocardial cells; however, during the reperfusion phase, excessive activation of autophagy can worsen myocardial cell damage and necrosis [40]. Cellular autophagy is regulated by various pathways, including the phosphatidylinositol-3-kinase (PI3K)/protein kinase B (Akt)/mammalian target of rapamycin (mTOR) signaling pathway, which has autophagy-inhibitory effects [41]. Several studies have shown that EA pretreatment may alleviate the degree of MI/RI by activating the PI3K/Akt/mTOR signaling pathway, thus inhibiting excessive autophagy in myocardial cells [42–44].

Microtubule-associated protein 1 light chain 3 (LC3) is an important autophagy marker protein, which exists in two forms: LC3-I and LC3-II. The increase in LC3-II expression reflects the enhanced autophagic activity within the cell [45]. Beclin-1 is a key protein that regulates and initiates the autophagy process, and its upregulation can induce cellular autophagy [46]. Zhang [47] found that EA pretreatment at Neiguan (PC6) downregulated the expression of LC3-II and Beclin-1 proteins, with the most significant effect observed on the seventh-day postischemia. This suggests that EA pretreatment can inhibit excessive activation of autophagy and protect myocardial tissue. Studies have shown that EA pretreatment, ischemic pretreatment, and moxibustion pretreatment can improve myocardial injury by reducing the expression levels of LC3-II and Beclin-1 proteins in rats, with EA pretreatment demonstrating the best effect in regulating myocardial autophagy [48, 49].

Mitochondrial autophagy is a form of autophagy wherein the body eliminates damaged mitochondria as a protective mechanism [50]. Mitochondria are the primary sites of ROS generation, and when mitochondrial autophagy is impaired, mitochondria cannot be removed in time, leading to excessive ROS production and triggering OS reactions [51]. Adenosine 5′-monophosphate–activated protein kinase (AMPK) is a protein kinase that maintains the energy balance of the body and can promote the activation of mitochondrial autophagy through various signaling pathways [52]. During myocardial I/R, an increase in AMPK expression enhances mitochondrial autophagy, effectively alleviating the inflammatory response and OS in myocardial cells [53]. Wang et al. [54] found that EA pretreatment at Neiguan (PC6) could reduce myocardial injury caused by acute myocardial ischemia, with the potential mechanism being the activation of the liver kinase B1 (LKB1)/AMPK/6-phosphofructo-2-kinase (PFK2) signaling pathway to promote mitochondrial autophagy. Studies have shown that activated AMPK can enhance intracellular autophagic levels by inhibiting mTOR [55]. Yin et al. [56] found that EA pretreatment combined with exercise pretreatment in rats with myocardial ischemia could increase the expression of AMPK in myocardial tissue while reducing the expression of mTOR. This study revealed that the combination of EA pretreatment and exercise pretreatment can promote autophagy by regulating the AMPK/mTOR signaling pathway, thereby alleviating the degree of MI/RI in rats.

Depression and CHD are both associated with impaired mitochondrial autophagy and OS [57]. The PTEN-induced kinase-1 (PINK-1)/Parkin signaling pathway is a classical pathway for mitochondrial autophagy. The PINK-1 and Parkin proteins play a crucial role in mitochondrial autophagy by identifying and marking damaged mitochondria to initiate the removal process [58]. It has been reported that EA therapy can enhance mitochondrial autophagy by promoting the expression of PINK-1 and Parkin proteins and reducing OS levels, thus providing therapeutic effects in rats with depression combined with the CHD model [59].

### 2.4. EA Inhibits Cell Apoptosis

Cell apoptosis is a programmed cell death process that plays a critical role in MI/RI. Activation of the cysteinyl aspartate–specific proteinase (caspase) family is considered a common endpoint in the various apoptotic pathways [60]. Caspase-3, as the terminal endonuclease of apoptosis, plays a key role in apoptosis together with its upstream regulator, caspase-9 [61]. Therefore, inhibiting the expression of caspase-3 and caspase-9 has become an important strategy to reduce myocardial cell apoptosis and protect heart function. Zhang et al. [62] found that after EA pretreatment, the gene expression of caspase-9 and caspase-3 in rat myocardial cells was significantly reduced. They proposed that the protective effect of EA pretreatment on myocardial I/R might be achieved by inhibiting myocardial cell apoptosis. In another experiment, the researchers observed that EA pretreatment reduced the expression levels of caspase-3 mRNA and LC3-II protein as well as the myocardial cell apoptosis index, suggesting that EA pretreatment could improve MI/RI by inhibiting excessive autophagy and reducing apoptosis. They also pointed out that further experimental studies are needed to explore the interaction between autophagy and apoptosis in MI/RI [63].

B-cell lymphoma-2 (Bcl-2) family molecules mainly regulate apoptosis through the mitochondrial pathway, with Bcl-2 being a representative antiapoptotic protein and Bcl-2-associated X protein (Bax) playing a proapoptotic role [64]. During myocardial ischemia, TRPV1 can promote the release of CGRP [65], upregulate Bcl-2 expression, and downregulate Bax and caspase-3 expression, thus reducing apoptosis [66]. Studies have shown that after EA pretreatment at Neiguan (PC6), the expression of TRPV1/CGRP pathway proteins and Bcl-2 protein significantly increased, while the expression levels of caspase-3 and Bax proteins and the cell apoptosis index significantly decreased. These results suggest that EA pretreatment can regulate apoptosis-related proteins through the TRPV1/CGRP pathway, reducing myocardial cell apoptosis and providing protection for ischemic myocardium [67].

Second mitochondria–derived activator of caspases (Smac)/direct inhibitor of apoptosis-binding protein with low pI (Diablo) can promote apoptosis by binding with the apoptosis inhibitory proteins (IAPs) to release the inhibition on caspases [68]. It has been found that after myocardial I/R, the expression levels of Smac/Diablo and caspase-9 significantly increased, while EA pretreatment significantly reduced the expression levels of Smac/Diablo and caspase-9. The researchers hypothesized that EA pretreatment alleviates MI/RI through inhibition of the Smac/Diablo-mediated caspase apoptosis pathway [69].

p38 mitogen-activated protein kinase (p38 MAPK), a member of the MAPK family, is activated in MI/RI and induces apoptosis. Mitogen-activated protein kinase kinase 3 (MKK3) and MKK6 are the upstream activators of the p38 MAPK pathway [70]. It has been reported that EA pretreatment inhibits the expression of MKK3, MKK6, and p38 MAPK; reduces Bax and caspase-3 expression; and increases Bcl-2 expression. These findings suggest that EA pretreatment may alleviate myocardial cell apoptosis by modulating the MKK3/MKK6–p38 MAPK signaling pathway [71].

Farnesoid X receptor (FXR) and small heterodimer partner (SHP) are members of the nuclear receptor superfamily. FXR regulates SHP gene transcription by binding to the FXR binding site in the SHP gene promoter region [72]. Studies have confirmed that the FXR/SHP signaling pathway plays a role in regulating apoptosis and is associated with MI/RI. Increased FXR expression can exacerbate myocardial cell apoptosis [73]. Evidence suggests that EA pretreatment significantly reduces infarct size and myocardial damage markers in I/R rats and effectively inhibits the gene expression of FXR and SHP in myocardial cells. This suggests that the protective effect of EA pretreatment on ischemic myocardium may be related to its regulation of the FXR/SHP signaling pathway [74]. Apoptosis-inducing factor (AIF) is a protein with apoptotic activity that promotes cell death upon apoptosis stimulation [75], while heat shock protein 70 (HSP70) is a well-known inhibitor of apoptosis, and its overexpression can prevent apoptosis triggered by various stimuli [76]. Liu et al. [77] found that EA pretreatment significantly reduced the expression levels of FXR, SHP, and AIF mRNA in myocardial tissue of MI/RI rats, while increasing the expression of HSP70 mRNA, thereby providing myocardial protection. They suggested that this protective effect may be related to the inhibition of the FXR/SHP signaling pathway–mediated myocardial cell apoptosis by EA pretreatment.

### 2.5. EA Inhibits Pyroptosis

Pyroptosis is a type of programmed cell death closely related to OS and inflammatory responses [78]. Pyroptosis not only plays a role in the MI/RI process but also plays a key role in the development of depression associated with CHD [79]. The classic pathway of pyroptosis is initiated when NLRP3 is activated, leading to the cleavage of procaspase-1 and the formation of active caspase-1. Caspase-1 then cleaves gasdermin D (GSDMD), resulting in cell membrane pore formation, release of cellular contents, and the induction of inflammation [80]. Studies have shown that upregulation of Nrf2 expression can alleviate inflammation by inhibiting the activation of NF-κB [81]. Xia [82] observed that EA pretreatment at Neiguan (PC6) could downregulate the expression of caspase-1 and GSDMD, thereby reducing infarct size and improving heart function in MI/RI rats. They proposed that the antipyroptotic mechanism of EA pretreatment might involve the regulation of the Nrf2–NF-κB signaling axis and the reduction of OS responses. Cui [83] found that after myocardial ischemia, EA at Ximen (PC4) could reduce myocardial injury by downregulating the mRNA and protein expression of TLR4, NF-κB p65, NLRP3, caspase-1, and GSDMD. This suggests that EA therapy may protect the heart by regulating the TLR4/NF-κB signaling pathway to inhibit pyroptosis. It is worth noting that this study observed that the reperfusion phase may be the optimal timing for EA therapy in treating AMI and speculated that the best intervention time for EA therapy to improve MI/RI may be during the ambulance transport following an AMI event. In addition, a study has indicated that EA therapy can treat depression associated with CHD by inhibiting caspase-1/GSDMD expression and reducing the release of inflammatory factors [84].

### 2.6. EA Regulates Gut Microbiota and Its Metabolites

The gut microbiota, consisting of microbial communities residing in the gut, participates in various physiological processes of the host. Changes in the structure of the gut microbiota and metabolic disturbances play a significant role in the development of dyslipidemia and AS, and they may also participate in the MI/RI process by affecting the metabolism of 5-hydroxytryptamine (5-HT) in the body. Metabolites produced by the gut microbiota are key mediators of the interaction between the microbiota and the host. Bile acids are important metabolic products of the gut microbiota and interact with the microbiota [85]. The enterohepatic circulation of bile acids is related to cholesterol metabolism, and cholesterol deposition accelerates AS. Therefore, abnormal bile acid metabolism can promote the development of AS [86]. Generally, the higher the bile acid content, the lighter the degree of AS lesions. The FXR/fibroblast growth factor 15 (FGF15)/FGF receptor 4 (FGFR4) pathway is an important signaling pathway in bile acid metabolism [87]. A group of researchers found that EA therapy could regulate the relative abundance of gut microbiota, upregulate host metabolites with therapeutic and preventive effects on AS, downregulate host metabolites that promote AS, and further improve host lipid metabolism, bile acid metabolism, and amino acid metabolism, thereby achieving a preventive and therapeutic effect on AS [88]. In subsequent studies, they revealed that EA therapy could correct gut microbiota metabolic disorders, inhibit the FXR/FGF15/FGFR4 pathway, and promote the expression of the rate-limiting enzyme in bile acid synthesis, CYP7A1, thereby enhancing bile acid secretion and effectively improving the pathological state of AS [89].

Trimethylamine N-oxide (TMAO) is an important metabolite of the gut microbiota that has been discovered in recent years. Increasing evidence suggests that TMAO promotes AS progression through several mechanisms, including affecting cholesterol metabolism, inducing OS, and exacerbating inflammation in the vascular walls [90]. CD36 is a key sensor and regulator of lipid metabolism [91], and TMAO can promote the expression of CD36, inhibiting cholesterol degradation and causing cholesterol accumulation within cells. This, in turn, activates the NLRP3 inflammasome and accelerates the progression of AS [92]. A recent study confirmed that EA therapy could alleviate arterial intima damage by regulating the TMAO/CD36/NLRP3 pathway, providing further experimental evidence for the use of EA in preventing and treating AS by modulating gut microbiota metabolites [93]. Furthermore, a similar study pointed out that EA at the Heart Meridian of Hand-Shaoyin acupoints can effectively regulate the structure, abundance, and diversity of gut microbiota; reduce TMAO levels; and improve myocardial ischemia [94].

5-HT is a widely distributed monoamine, and its metabolic abnormalities can lead to the onset of cardiovascular diseases. The gut microbiota can stimulate enterochromaffin (EC) cells to synthesize and secrete 5-HT [95]. I/R can cause changes in the gut microbiota distribution, leading to abnormal 5-HT metabolism and inducing or exacerbating myocardial injury [96]. One study showed that EA pretreatment effectively alleviated myocardial injury in I/R model rats, and its mechanism of action may be related to regulating the gut microbiota, inhibiting the secretion of 5-HT by EC cells, reducing 5-HT and related metabolites in the ileum and serum, and lowering myocardial 5-HT levels and its receptor expression [97].

### 2.7. EA Affects the Nervous System

In recent years, researchers have focused on exploring the specific mechanisms by which EA therapy exerts myocardial protection through its effects on the nervous system. Glutamatergic neurons are the primary excitatory neurons in the central nervous system and play a role in cardiovascular regulation by influencing the autonomic nervous system. Zhang et al. [98] clarified that EA therapy exerts its protective effects on ischemic myocardium and reduces the occurrence of ventricular premature beats by lowering the activity of glutamatergic neurons in layer 5 of the primary motor cortex and regulating cardiac autonomic balance. Shu [99] found that EA pretreatment regulates sympathetic nerve excitability by mediating changes in the activity of glutamatergic neurons in the lateral hypothalamic area (LHA), improving MI/RI. Furthermore, their study revealed that the LHA-fastigial nucleus (FN) neural pathway participates in the EA pretreatment process that alleviates MI/RI.

Substance P (SP) is an important substance in cardiovascular regulation. It not only increases the discharge frequency of low-level central sympathetic neurons but also activates nitric oxide synthase (NOS) activity, which enhances nitric oxide (NO) production, thus contributing to myocardial protection in collaboration with NO. Li [100] discovered that EA at Neiguan (PC6) could reduce the discharge frequency of the vagus nerve and increase the discharge frequency of the sympathetic nerve in AMI rats, improving myocardial blood supply. Further observation revealed that these effects were related to the increased expression of SP and NOS in the paraventricular nucleus (PVN) of the hypothalamus and the lateral horn of the spinal cord, which were induced by EA at Neiguan (PC6).

Myocardial I/R causes sympathetic nerve regeneration and an increase in sympathetic nerve density, leading to sympathetic nerve remodeling that lowers cardiac electrical stability and contributes to ventricular remodeling, which can ultimately result in life-threatening arrhythmias and sudden death [101]. Growth-associated protein 43 (GAP-43) is considered a marker of nerve growth, and its expression is positively correlated with sympathetic nerve excitability [102]. Tyrosine hydroxylase (TH) is a major marker of sympathetic nerve regeneration in the heart [103]. Fu [104] found that EA pretreatment could inhibit the excessive expression of GAP-43 and optimize the distribution density of TH-positive fibers, thereby suppressing excessive sympathetic nerve regeneration in the I/R region and correcting the uneven distribution of sympathetic nerve density in damaged myocardium. This process enhanced cardiac electrical stability, reduced ventricular remodeling, and ultimately protected cardiac function. Another study also confirmed that EA therapy might protect the heart by lowering the expression levels of TH and GAP-43 proteins in the myocardial tissue of AMI mice, thus inhibiting excessive sympathetic nerve excitation postinfarction [105].

Both the PVN of the hypothalamus and the nucleus tractus solitarius (NTS) are involved in regulating defensive cardiovascular responses. Research has shown that EA therapy can effectively alleviate MI/RI by regulating the functions of the PVN and NTS [106]. Modulating neuronal electrical activity in the PVN may be one of the key central mechanisms through which EA therapy exerts its anti-MI/RI effects [107]. In addition, beta-endorphins and IL-1 in the PVN may be key central effectors responsible for the antiacute myocardial ischemia effects of EA at Neiguan (PC6) [108].

### 2.8. EA Affects Platelet Activation and Aggregation

Platelet activation and aggregation are crucial factors in thrombosis formation and are closely associated with CHD. Granule membrane protein-140 (GMP-140) is one of the specific markers for platelet activation, promoting the adhesion of leukocytes to platelets and their aggregation at the site of thrombosis [109]. Platelet-activating factor (PAF) has a strong effect on promoting platelet aggregation and release, thereby facilitating thrombosis formation [110]. Huang [111] found that EA at Neiguan (PC6) can reduce the excessive elevation of GMP-140 and PAF levels in serum during AMI, inhibit platelet activation, and reduce the adhesion of leukocytes and platelets, and their aggregation at the thrombus site, thereby decreasing the likelihood of recurrent embolism. Another study also confirmed that EA at Neiguan (PC6) could lower PAF levels in the serum of rabbits with acute myocardial ischemia, thereby alleviating ischemic myocardial tissue damage [112]. NO is an endogenous vasodilator that inhibits platelet adhesion and aggregation [113]. A group of researchers observed that EA at Neiguan (PC6) could reduce GMP-140 levels in the plasma of rabbits with acute myocardial ischemia while increasing NO levels. Therefore, they concluded that EA therapy may exert its protective effects on ischemic myocardium by reducing platelet activation and minimizing thrombus formation [114].

### 2.9. EA Reduces Calcium (Ca^2+^) Overload

During myocardial I/R, the release of Ca^2+^ stored in the endoplasmic reticulum (ER)/sarcoplasmic reticulum (SR) within myocardial cells leads to an increase in cytosolic-free Ca^2+^ concentrations. This phenomenon of Ca^2+^ overload is a major factor in MI/RI [115]. Calmodulin (CaM) is one of the most important calcium receptor proteins within cells [116]. L-type calcium channels (LTCCs) are the main pathway for extracellular Ca^2+^ influx during myocardial cell excitation [117]. The Ca^2+^/CaM complex regulates Ca^2+^/calmodulin-dependent protein kinase II (CaMK II), which in turn modulates LTCCs' activity, resulting in prolonged channel open times and increased Ca^2+^ influx, ultimately causing cellular Ca^2+^ overload [118]. A study found that EA at Neiguan (PC6) could significantly reduce the gene expression of CaM, CaMK II, and LTCCs in ischemic myocardial tissue. This suggests that EA therapy for myocardial ischemia may alleviate Ca^2+^ overload by modulating LTCCs [119].

Calreticulin (CRT) is a major Ca^2+^ binding protein in the ER and SR, involved in maintaining intracellular Ca^2+^ homeostasis. Upregulation of its expression plays a significant role in reducing Ca^2+^ overload [120]. Tian et al. [121] found that EA pretreatment at Neiguan (PC6) or Ximen (PC4) could reduce intracellular Ca^2+^ overload in myocardial cells by upregulating CRT mRNA expression, thereby protecting ischemic myocardium.

Sarco/ER Ca^2+^-ATPase (SERCA) is an important protein involved in Ca^2+^ regulation, primarily responsible for pumping Ca^2+^ from the cytoplasm into the SR, which lowers cytosolic Ca^2+^ levels and increases the SR's Ca^2+^ reserves, ensuring normal physiological activity of myocardial cells [122]. Myocardial ischemia reduces SERCA activity, promoting Ca^2+^ overload, leading to myocardial injury and decreased cardiac function [123]. Ren et al. [124] found that EA at Neiguan (PC6) and Gongsun (SP4) can increase SERCA activity and inhibit the onset of Ca^2+^ overload in myocardial cells, thus protecting damaged myocardial tissue. Another study demonstrated that EA therapy could upregulate SERCA mRNA expression and enhance SERCA activity, strengthening the SR's ability to take up Ca^2+^, thereby reducing the damage caused by Ca^2+^ overload in I/R myocardial tissue [125].

### 2.10. EA Stabilizes the Cytoskeleton

The cytoskeleton plays a crucial role in maintaining the structure and function of myocardial cells. After myocardial ischemia and hypoxia, the cytoskeleton can exhibit varying degrees of damage [126]. The cytoskeleton is composed of polymers of actin, microtubule proteins, and intermediate filament proteins [127]. Desmin, an intermediate filament protein of the cytoskeleton, and alpha-actinin, an actin-binding protein, are involved in maintaining the integrity of myocardial cells and regulating myocardial contraction [127, 128]. A study has confirmed that EA pretreatment can upregulate the expression of desmin and alpha-actinin, alleviating myocardial cell damage during I/R and providing protection to the heart [129].

### 2.11. EA Affects Myocardial Electrical Activity

The transient outward potassium current (Ito) channel plays a crucial role in the repolarization of the myocardial action potential and maintaining the stability of the cardiac electrical activity. Its channel proteins are mainly encoded by the Kv1.4, Kv4.2, and Kv4.3 genes [130]. Kchip2 is a component of the Kv4.2/Kv4.3 channel protein complex [131]. The electrophysiological remodeling caused by myocardial ischemia is often associated with the suppression of Ito due to decreased expression of its channel proteins [130]. Sa et al. [132] proposed that EA therapy can improve myocardial electrical remodeling in rats with AMI, reducing the risk of arrhythmias post-AMI. The mechanism is believed to be related to the upregulation of Kv4.2 and Kchip2 expression, which in turn promotes the opening of the Ito channel. Wang et al. [133] also suggested that the increased opening of the Ito channel is one of the mechanisms through which EA therapy improves myocardial ischemia. In their study, the researchers found that EA at Neiguan (PC6), Lieque (LU7), and Zusanli (ST36) in myocardial ischemia mice enhanced the expression of Kv1.4, Kv4.2, Kv4.3, and Kchip2 in myocardial cells, with the most significant increase observed in the Neiguan (PC6) group.

### 2.12. EA Promotes Angiogenesis and Myocardial Tissue Regeneration

Vascular endothelial growth factor (VEGF) is a potent angiogenic factor that exhibits significant mitogenic and antiapoptotic effects on endothelial cells [134]. Studies have shown that EA therapy can significantly improve heart function after acute myocardial ischemia and increase long-term survival rates. Its mechanism of action may be related to EA's ability to upregulate VEGF expression in cardiomyocytes, promote myocardial angiogenesis, and accelerate the establishment of collateral circulation in ischemic peripheral tissues [135, 136]. Hypoxia-inducible factor-1 alpha (HIF-1 alpha) plays a central role in myocardial tissue oxygen balance regulation. Its stability is controlled by histone deacetylase 5 (HDAC5) [137, 138], and the activation of HDAC5 is regulated by AMPK [139]. HIF-1 alpha promotes myocardial vascular generation and reduces ischemic myocardial damage by increasing VEGF expression [140]. Zhou [141] found that EA at the Pericardium Meridian of Hand–Jueyin acupoints can promote the expression of HIF-1 alpha and VEGF in the serum and ischemic myocardium of rats with myocardial ischemia, suggesting that this is one of the mechanisms of its antimyocardial ischemia effect. Lu et al. [142] found that EA therapy can activate the AMPK–HDAC5–HIF-1 alpha signaling cascade, thereby promoting VEGF expression, aiding in angiogenesis, and reducing the infarcted myocardial area. Activation of the AMPK/Kruppel-like factor 2 (KLF2) signaling pathway also promotes VEGF expression [143]. A study has confirmed that EA at Neiguan (PC6) can exert myocardial protective effects, potentially through the activation of the AMPK/KLF2 signaling pathway, upregulation of VEGF expression, promotion of myocardial angiogenesis, and improvement of myocardial microcirculation in rats with MI/RI [144]. Another study confirmed that EA pretreatment can enhance VEGF levels in serum and increase VEGF protein expression in the ischemic myocardium, thereby providing cardioprotective effects in chronic myocardial ischemia rats [145].

Stem cell factor (SCF) plays a significant role in promoting the differentiation of stem cells to cardiomyocytes upon homing to the myocardium [146]. Research has shown that both pre-MI EA pretreatment and post-MI EA therapy can significantly increase the levels of SCF in peripheral blood and myocardial tissue of MI rats, accelerate the migration of circulating stem cells to ischemic areas, and promote myocardial tissue regeneration [147]. Notably, early EA therapy after MI can more effectively enhance the mobilization of endogenous stem cells, thereby improving prognosis [147]. Stromal cell–derived factor-1 (SDF-1) is a chemotactic factor that binds to its receptor, C-X-C chemokine receptor 4 (CXCR4). The SDF-1/CXCR4 signaling pathway plays an essential role in promoting angiogenesis and the mobilization of stem cells to infarcted areas after myocardial ischemia [148]. C-kit, the receptor for SCF, exhibits an increased number of positive cells in the heart, which effectively promotes myocardial repair and angiogenesis [149]. Research has shown that EA at Neiguan (PC6) and Xinshu (BL15) can effectively repair damaged myocardium and reduce infarcted area, likely through the mobilization of endogenous stem cells mediated by the SDF-1/CXCR4 and SCF/c-kit pathways [150]. In addition, the study also found that EA therapy can improve the local microenvironment after AMI and proposed that EA therapy combined with stem cell transplantation may represent a novel treatment strategy for AMI [150].

### 2.13. EA Affects Transcriptomics and Metabolomics

Several researchers have explored the mechanisms by which EA therapy and EA pretreatment exert cardioprotective effects in ischemic myocardium from the perspectives of transcriptomics and metabolomics. A study has revealed that multiple genes and pathways associated with signal transduction, immune regulation, and energy metabolism are related to MI/RI [151]. This study further confirmed that EA pretreatment at Jiaji (EX–B2) regulates the expression of multiple genes and pathways in MI/RI rats, including extracellular matrix receptor interaction, focal adhesion, and chemokine signaling pathways, which may contribute to the cardioprotective effects of EA pretreatment [151]. I/R injury causes significant changes in the serum metabolic profiles of rats [152]. Experiments have demonstrated that EA pretreatment at Neiguan (PC6) may exert cardioprotective effects in I/R rats by regulating multiple metabolic pathways, including glucose metabolism, pyruvate metabolism, amino acid metabolism, and ketone body metabolism [153, 154]. Wu et al. [155] performed the untargeted metabolic profiling analysis of the prefrontal cortex in acute myocardial ischemia rats after EA therapy. The results showed that EA therapy could reverse the levels of N-acetylasparagine, sphingosine, and dihydrosphingosine. Consequently, they concluded that the improvement in myocardial ischemia by EA therapy may be achieved through the regulation of sphingolipid metabolic pathways in the prefrontal cortex. Xuan [150] suggested that the mechanism of EA therapy in treating AMI may be related to the regulation of taurine, hypotaurine, and tryptophan metabolism pathways. Another study found that EA therapy can improve myocardial ischemia by regulating glycerophospholipid metabolism, bile acid metabolism, arachidonic acid metabolism, linoleic acid metabolism, and sphingolipid metabolism, providing additional experimental evidence for the effectiveness of EA therapy [156].

In summary, EA therapy has been shown to exert therapeutic effects on CHD through various mechanisms, such as alleviating inflammation and OS, regulating autophagy levels, inhibiting apoptosis and pyroptosis, and modulating gut microbiota and metabolites. These studies provide valuable references for the application of EA therapy in the treatment of CHD patients. However, further in-depth research is needed to clarify the potential interconnections between these mechanisms and how they work together to exert therapeutic effects (Table 1).

## 3. The Role of EA in the Treatment of Patients With CHD

### 3.1. Effects of EA on Patients With Angina Pectoris

Angina pectoris is a common symptom of CHD, and the effectiveness and safety of EA therapy in treating angina pectoris have been clinically validated. Huang et al. [157] found that, compared to the Compound Danshen Dropping Pill group, the EA at Neiguan (PC6) group exhibited significantly superior outcomes in alleviating angina pectoris symptoms, enhancing electrocardiogram efficacy, reducing the frequency of angina attacks, and decreasing nitroglycerin usage, with good safety. Chen et al. [158] revealed that a treatment regimen combining the oral Compound Danshen Dropping Pill with EA at Neiguan (PC6), Danzhong (CV17), and Xinshu (BL15) showed more superior effects in improving angina symptoms and enhancing clinical efficacy compared to medication alone. Xu et al. [159] showed that combining EA at Neiguan (PC6) and Ximen (PC4) with Western medicine significantly improved the capacity to carry out basic activities of daily living, physical symptoms, emotional status, and quality of life in angina pectoris patients.

Apolipoprotein A1 (Apo-A1), the principal structural protein of high-density lipoprotein cholesterol (HDL-C), exerts a protective effect against AS. Apolipoprotein B (Apo-B), which is primarily found in low-density lipoprotein cholesterol (LDL-C), plays an important role in promoting the formation of AS. The ratio of Apo-A1/Apo-B < 1 is considered a risk indicator for cardiovascular diseases. Zou et al. [160] found that EA at Neiguan (PC6) positively influenced apolipoprotein levels in myocardial ischemia patients, specifically by increasing Apo-A1 levels, decreasing Apo-B levels, and elevating the Apo-A1/Apo-B ratio, demonstrating the effectiveness of EA therapy in regulating serum apolipoprotein levels and preventing coronary AS.

Coronary artery calcification is a marker of coronary AS, and there is a linear relationship between the degree of calcification and plaque progression [161]. Coronary artery calcification score (CCS) is an independent predictor of coronary artery events [162]. Zhang et al. [163] found that EA at Neiguan (PC6) and Tongli (HT5), or Kongzui (LU6) and Taiyuan (LU9), could reduce the pain level of angina pectoris patients and the nitroglycerin dose, while significantly decreasing CCS. However, EA at Neiguan (PC6) and Tongli (HT5) showed more significant therapeutic effects compared to Kongzui (LU6) and Taiyuan (LU9). These findings provide clinical evidence for the hypothesis that the choice of acupoints along the meridians can maximize therapeutic effects.

The occurrence of angina pectoris is related to abnormalities in blood rheology properties, with an increased blood viscosity reflecting the severity of myocardial ischemia. Reduced erythrocyte deformability and enhanced erythrocyte aggregation are the main causes of increased blood viscosity and reduced blood fluidity. Stroke volume (SV), cardiac output (CO), cardiac index (CI), and ejection fraction (EF) are important parameters for evaluating heart function. Huang et al. [164] found that EA at Neiguan (PC6) and Ximen (PC4) combined with oral Western medicine significantly improved erythrocyte aggregation ability, whole blood viscosity, and plasma viscosity, while enhancing erythrocyte deformability more effectively than medication alone. Furthermore, a comparable study has observed that, compared to conventional drug treatment, the combination of EA at Neiguan (PC6), Yunmen (LU2), and Lieque (LU7) with conventional drug treatment significantly improved SV, CO, and erythrocyte deformability index, while significantly reducing erythrocyte aggregation index and plasma viscosity. These findings suggest that EA combined with drug therapy positively influences heart function and blood circulation in angina pectoris patients [165]. Tong et al. [166] divided angina pectoris patients into two groups: one receiving conventional Western medicine therapy alone, while the other receiving EA at Neiguan (PC6) and Ximen (PC4) combined with conventional Western medicine. The results showed that both groups had significant improvements in SV, CO, CI, and EF, but the EA combined with the Western medicine therapy group showed superior results, strongly supporting that EA combined with conventional Western medicine enhances myocardial contractility, increases coronary blood flow, and improves CO, thereby improving heart function in patients.

The neutrophil-to-lymphocyte ratio (NLR) has been identified as an independent predictor for the prognosis of CHD patients. A reduction in NLR suggests an improvement in the degree of AS and a reduction in angina symptoms in CHD patients. Wang et al. [167] found that patients receiving EA at Neiguan (PC6) combined with conventional medication therapy had significantly lower NLR values than those receiving medication alone, indicating that EA therapy improves the prognosis of CHD patients.

Heart rate recovery (HRR) values, as a quantitative indicator of heart rate (HR), decline across various postexercise time points and provide a direct reflection of the heart's autonomic nervous system regulation. Low HRR values often indicate poor prognosis and decreased quality of life in cardiovascular disease patients. In addition, exercise capacity is an important factor affecting the quality of life in CHD patients. Generally, the greater a patient's exercise capacity, the better their quality of life. Aerobic exercise is currently the main method of cardiac rehabilitation. Zhou [168] found that combining EA at Neiguan (PC6) and Ximen (PC4) with aerobic exercise improved the overall coordination of autonomic nervous function, reduced OS levels, and enhanced HRR values and exercise capacity more effectively than aerobic exercise alone. This study provides new treatment approaches for the rehabilitation of CHD patients.

An increasing number of studies are focusing on the effects of EA therapy on the electrocardiogram (ECG) and HR. Changes in the ST segment and T-wave of the ECG are crucial for evaluating the degree and location of myocardial ischemia. Studies have shown that EA at Shenmen (HT7) and Shaohai (HT3) significantly lowered heart rate and increased T-wave amplitude. Among them, EA at Shenmen (HT7) significantly improved the ST segment, while EA at Shaohai (HT3) had no significant effect on the ST segment [169]. Despite this, both acupoints effectively improved myocardial ischemia. However, no significant synergistic effect was observed when both acupoints were used simultaneously, possibly because the individual stimulation already reached or neared the therapeutic threshold, and combining them did not further enhance the effect. In addition, the study found that the acupuncture effect varied over time, peaking 30 min after acupuncture and gradually diminishing, with the effect almost disappearing 60 min later [169]. Su [170] conducted a detailed study on the differences in the effects of EA at Neiguan (PC6) and Ximen (PC4) on ECG ST segment and T-wave improvement in angina pectoris patients. The results showed that EA at Neiguan (PC6) exhibited stronger efficacy and higher specificity in improving myocardial ischemia compared to EA at Ximen (PC4). Yang et al. [171] conducted ECG tests before and after EA therapy on 60 stable angina patients. The results indicated that EA at Lingtai (GV10) and Shendao (GV11) showed comparable effects in improving ST segment performance across both groups. The enhancement in T-wave amplitude was more pronounced in the V4, V5, and V6 leads compared to the II, III, and avF leads, concluding that EA at Lingtai (GV10) and Shendao (GV11) improved ischemia in the inferior and anterior walls of the myocardium, with a more significant effect on the anterior wall. A similar study has indicated that EA at Neiguan (PC6) significantly improved myocardial ischemia in both the inferior and anterior walls of stable angina patients, with a particularly marked improvement in the anterior wall ischemia [172].

### 3.2. Effects of EA on Patients Undergoing Percutaneous Coronary Intervention (PCI)

PCI is an effective treatment for CHD, significantly improving patient prognosis and quality of life. However, it inevitably results in MI/RI. Numerous foundational experimental studies have shown that EA therapy can effectively alleviate MI/RI, with these effects being well-validated in post-PCI patients. Zeng [173] conducted a study on CHD patients, applying EA at Neiguan (PC6), Daling (PC7), and Taiyuan (LU9) 2 days before and 14 days after PCI. The results demonstrated that EA therapy effectively improved myocardial ischemia in post-PCI patients, increased exercise tolerance, significantly reduced angina symptoms, alleviated anxiety, improved heart function, controlled blood pressure fluctuations, and reduced the occurrence of arrhythmias. This study provides valuable clinical evidence for the application of EA therapy in patients undergoing PCI. In a prospective, randomized, multicenter clinical trial, researchers found that EA pretreatment at Neiguan (PC6) and Ximen (PC4) 1-2 h before PCI significantly reduced early myocardial injury, lowered the level of an early inflammatory response, and improved early myocardial metabolism. Furthermore, during the 24-month follow-up period post-PCI, EA attenuated continued to show positive effects on cardiac function and significantly reduced the incidence of major cardiovascular adverse events (MACEs) [174]. These findings highlight the potential beneficial role of EA therapy in improving both the short- and long-term prognosis of post-PCI patients.

A major complication after PCI is in-stent restenosis (ISR), which, despite advancements in stent technology, still has a high incidence and severely impacts patient prognosis [175]. Current research suggests that ISR primarily results from excessive proliferation and migration of vascular smooth muscle cells (SMCs) due to arterial damage, with inflammation playing a key role in this process. This leads to coronary vascular remodeling and in-lumen restenosis [176]. S100 calcium-binding protein A4 (S100A4) has been identified as a biomarker for restenotic coronary SMCs, where it is highly expressed in SMCs involved in stent-induced intimal thickening, promoting their proliferation and migration [177]. In addition, S100A4 can enhance the inflammatory response by inducing the expression of chemotactic factors and adhesion molecules in vascular endothelial cells [178]. A study found that EA at Neiguan (PC6) and Ximen (PC4) effectively reduced the incidence of ISR, serum S100A4 levels, and the occurrence of MACE after PCI, while also prolonging patient survival. Researchers speculated that EA therapy might reduce the occurrence of ISR and improve the prognosis of CHD patients by lowering serum S100A4 levels, which could suppress vascular inflammation and delay the proliferation of SMCs. This suggests that the potential mechanism for reducing ISR and improving prognosis may involve the suppression of inflammatory responses and SMCs' proliferation [179].

### 3.3. Effects of EA on Patients Undergoing Coronary Artery Bypass Grafting (CABG)

CABG is one of the key strategies for treating CHD, providing effective revascularization. However, it is often accompanied by risks of MI/RI as well as severe complications.

High-sensitivity C-reactive protein (hs-CRP) and cardiac troponin I (cTnI) are commonly used to reflect cardiovascular inflammation and cardiac tissue damage. Mu et al. [180] found that in the EA pretreatment group after CABG, the increase in cTnI and hs-CRP levels, as well as the occurrence of postoperative asymptomatic myocardial ischemia and arrhythmias, was significantly lower compared to the non-EA pretreatment group. However, there was no significant difference in improving heart function between the two groups. This suggests that EA pretreatment offers a certain degree of myocardial protection for post-CABG patients and can effectively reduce the occurrence of adverse cardiac events.

**Figure 1:**
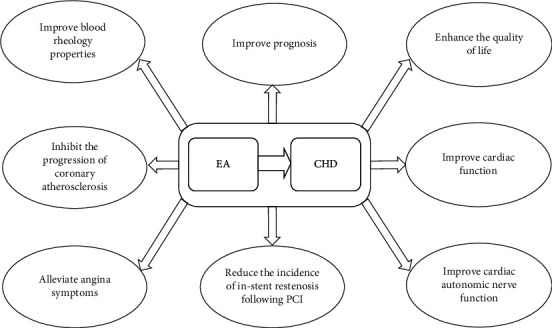
Effects of EA therapy on patients with CHD.

Cardiac dysfunction is one of the most common complications after CABG, negatively impacting the patient's quality of life and safety. Generally, cardiac dysfunction leads to varying degrees of decline in CO, CI, and SV, while central venous pressure (CVP), pulmonary artery pressure (PAP), pulmonary capillary wedge pressure (PCWP), and mean arterial pressure (MAP) tend to increase. HR changes are also closely related to heart function. Within a certain range, an increased HR can enhance myocardial contractility and CO. During the same period, both sets of researchers employed floating catheter technology to monitor heart function–related indicators to evaluate the effects of EA at Neiguan (PC6) and Shenmen (HT7) on patients receiving conventional treatment after CABG [181, 182]. One study found that, compared to the control group, only HR showed significant improvement in the EA treatment group, while CI, CO, MAP, and PCWP showed no significant changes [181]. Notably, this study only conducted intergroup comparisons between the control and experimental groups without analyzing the intragroup pre- and posttreatment changes. In contrast, another study compared changes in hemodynamic parameters both within the groups before and after treatment and between the two groups. The results showed that in the EA treatment group, CVP, PAP, and PCWP significantly decreased compared to the pretreatment levels, while CO, CI, and SV significantly increased. Moreover, the improvement in these parameters in the EA treatment group was greater than in the control group [182]. Overall, EA therapy demonstrated a positive role in improving cardiac function in post-CABG patients (Figure 1).

## 4. Discussion

EA therapy, a treatment method based on traditional acupuncture combined with modern electrotherapy, has the advantage of being simple, with minimal adverse reactions. Through a review of the literature, we found that the feasibility and efficacy of EA therapy as an adjunctive treatment for CHD have been confirmed, but several issues remain that warrant further exploration. One challenge is that EA therapy requires patients to tolerate both acupuncture and electrical stimulation. For patients who are sensitive to pain or have a fear of acupuncture needles, it may be difficult for them to accept this treatment. Therefore, healthcare providers need to ensure that these patients are fully informed about the principles and processes of EA therapy before treatment, providing necessary psychological support to alleviate fear and anxiety. In addition, gradually increasing the intensity of acupuncture may help patients adjust and make the treatment process smoother. In clinical practice, ensuring the safety of EA therapy should be a primary concern for healthcare providers. To meet clinical needs and improve the safety of EA therapy, it is crucial to develop a safer EA apparatus. Furthermore, healthcare professionals must strictly follow operational protocols when using the EA apparatus to ensure patient safety. As a common cardiovascular disease, CHD is often comorbid with other conditions. Currently, research on EA therapy for CHD comorbidities is limited to studies focusing on anxiety and depression in CHD patients. There is a lack of studies on other comorbidities, suggesting that there are many potential research directions in this field that warrant further exploration. Some researchers have explored the impact of acupuncture timing, duration, and acupoint selection on CHD efficacy. However, the influence of electrical pulse waveform, frequency, and amplitude on CHD outcomes remains unclear and requires further investigation. This highlights a lack of standardized protocols in EA therapy for CHD. Therefore, the development of standardized quantitative guidelines for EA therapy of CHD and the establishment of a regulated treatment system should be a focal point for future research. More critically, there is a lack of comparative studies between EA therapy and traditional acupuncture for treating CHD. Specifically, the role of electrical pulses in EA therapy remains ambiguous, which is an important issue for future research. EA therapy, as a physiotherapy method based on TCM theory, currently lacks research on acupuncture point selection for different TCM syndromes in CHD. Future studies could focus on an in-depth exploration of this field to further refine and optimize EA treatment protocols. A series of animal studies have preliminarily examined the potential mechanisms of EA therapy in preventing and treating CHD at the microlevel. Future research should continue to deepen the mechanistic study of EA therapy in the prevention and treatment of CHD, providing more objective evidence and theoretical support for its use. Although numerous basic studies have demonstrated that EA pretreatment can effectively prevent and treat MI/RI through various mechanisms, the clinical reality is that AMI often occurs suddenly and unpredictably, making it difficult to implement EA interventions before acute myocardial ischemia. Therefore, future research should focus on exploring how to conduct preventive EA interventions before the onset of AMI, as well as the impact of the timing and duration of EA on treatment efficacy. In addition, considering the individual differences in CHD patients, future studies could explore personalized EA treatment plans to better meet patients' therapeutic needs. Despite the challenges in applying EA therapy to CHD treatment, it is believed that with the development of more scientifically rigorous research, EA therapy will play an increasingly prominent role in the treatment of CHD.

## Figures and Tables

**Table 1:** Relevant signaling pathways research of EA therapy for CHD.

References	Effects of EA on relevant signaling pathways	EA intervention parameters
Wu [11]	Enhancing the expression of the TRPV1/CGRP signaling pathway to alleviate inflammatory responses	Neiguan (PC6), 2 Hz/100 Hz, 2 mA, 20 min, once a day, 7 days
Shao et al. [29]	Activating the Nrf2–ARE signaling pathway to upregulate HO-1 gene expression, thereby reducing oxidative stress	T4-T5 Jiaji (EX-B2), 2 Hz/100 Hz, 1 mA, 30 min, once a day, 7 days
Zhang et al. [42]	Activating the PI3K/Akt/mTOR signaling pathway, thus inhibiting excessive autophagy in cardiomyocytes	Neiguan (PC6) and Zusanli (ST36), 2 Hz, 1 mA, 20 min, once a day, 7 days
He et al. [43]	Activating the PI3K/Akt signaling pathway, which consequently attenuates autophagic activity in cardiomyocytes	Neiguan (PC6), 10 Hz/50 Hz, 1-2 mA, 30 min, once a day, 7 days
Liu [44]	Activating the Akt/mTOR signaling pathway, thereby inhibiting excessive autophagy in cardiomyocytes	Neiguan (PC6), 10 Hz/50 Hz, 1-2 mA, 30 min, once a day, 7 days
Wang et al. [54]	Activating the LKB1/AMPK/PFK2 signaling pathway, thereby promoting mitochondrial autophagy in myocardial tissue	Neiguan (PC6), 2 Hz/15 Hz, 1 mA, 30 min, once a day, 14 days
Yin et al. [56]	Regulating the AMPK/mTOR signaling pathway to activate AMPK and inhibit mTOR, thereby promoting mitochondrial autophagy in myocardial tissue	Xinshu (BL15) and Shenmen (HT7), 2 Hz, 3 V, 20 min/10 min, once a day, 6 times/week, 3 weeks
Zhou [59]	Activating the PINK-1/Parkin signaling pathway in the hippocampus, thereby promoting mitochondrial autophagy	Baihui (GV20), Neiguan (PC6), Zusanli (ST36), Sanyinjiao (SP6), and Taichong (LR3), 2 Hz, 0.6 mA, 15 min, once a day, 21 days
Wu et al. [67]	Activating the TRPV1/CGRP signaling pathway to increase Bcl-2 protein levels and decrease the expression of Bax and caspase-3 proteins, thus alleviating myocardial cell apoptosis	Neiguan (PC6), 2 Hz/100 Hz, 2 mA, 20 min, once a day, 7 days
Zhang et al. [69]	Inhibiting the Smac/Diablo–caspase apoptotic pathway and reducing the gene expression levels of Smac/Diablo and caspase-9, thereby alleviating cardiomyocyte apoptosis	Neiguan (PC6), Guanyuan (CV4), and Zusanli (ST36), 2 Hz, 1 mA, 20 min, once a day, 7 days
Chen et al. [71]	Regulating the MKK3/MKK6-p38 MAPK signaling pathway and downregulating the mRNA expression of MKK3, MKK6, and p38 MAPK, thereby inhibiting apoptosis in cells within the myocardial infarction area	Neiguan (PC6), Guanyuan (CV4), and Zusanli (ST36), 2 Hz, 1 mA, 20 min, once a day, 7 days
Li et al. [74]	Inhibiting the FXR/SHP signaling pathway, thus alleviating cardiomyocyte apoptosis	Neiguan (PC6), Guanyuan (CV4), and Zusanli (ST36), 2 Hz, 1 mA, 20 min, once a day, 7 days
Liu et al. [77]	Inhibiting the FXR/SHP signaling pathway, thereby suppressing cardiomyocyte apoptosis	Neiguan (PC6), Guanyuan (CV4), and Zusanli (ST36), 2 Hz/100 Hz, 1 mA, 20 min, once a day, 7 days
Xia [82]	Regulating the Nrf2–NF-κB signaling pathway to upregulate Nrf2 protein expression and downregulate NF-κB p65 protein expression, thus reducing cardiomyocyte pyroptosis	Neiguan (PC6), 2 Hz/100 Hz, 2 mA, 20 min, once a day, 3 days
Cui [83]	Inhibiting the TLR4/NF-κB signaling pathway to downregulate the mRNA and protein expression of TLR4, NF-κB p65, and NLRP3, thereby suppressing the pyroptosis of cardiomyocytes	Ximen (PC4), 2 Hz, 30 min
Liu [89]	Inhibiting the FXR/FGF15/FGFR4 pathway, promoting the mRNA and protein expression of CYP7A1, thereby improving atherosclerosis	Neiguan (PC6), Xinshu (BL15), and Geshu (BL17), 2 Hz/15 Hz, 0.3 mA, 10 min, once every other day, 11 weeks
Lu et al. [142]	Activating the AMPK–HDAC5–HIF-1 alpha signaling pathway to upregulate the mRNA and protein expression of VEGF, thereby promoting angiogenesis in the myocardium	Neiguan (PC6), 2 Hz/15 Hz, 1.5–2 mA, 30 min, once a day, 4 days
Liu et al. [144]	Activating the AMPK/KLF2 signaling pathway to upregulate the protein expression of VEGF and VEGFR2, thereby promoting angiogenesis in the myocardium	Neiguan (PC6), 2 Hz/100 Hz, 2 mA, 20 min, once a day, 5 days
Xuan [150]	Activating the SDF-1/CXCR4 signaling pathway to promote the migration of endogenous stem cells to the injury site, thus protecting theischemic myocardium	Neiguan (PC6) and Xinshu (BL15), 20 Hz, 1 mA, 30 min, once a day, 6 times/week, 4 weeks

Abbreviations: Akt = protein kinase B; AMPK = adenosine 5′-monophosphate–activated protein kinase; ARE = antioxidant response element; Bax = Bcl-2-associated X protein; Bcl-2 = B-cell lymphoma-2; caspase-3 = cysteinyl aspartate–specific proteinase-3; caspase-9 = cysteinyl aspartate–specific proteinase-9; CGRP = calcitonin gene–related peptide; CXCR4 = C-X-C chemokine receptor 4; CYP7A1 = cholesterol 7 alpha-hydroxylase; Diablo = direct inhibitor of apoptosis-binding protein with low pI; FGFR4 = fibroblast growth factor receptor 4; FGF15 = fibroblast growth factor 15; FXR = farnesoid X receptor; HDAC5 = histone deacetylase 5; HIF-1 alpha = hypoxia-inducible factor-1 alpha; KLF2 = Kruppel-like factor 2; LKB1 = liver kinase B1; MAPK = mitogen-activated protein kinase; MKK3 = mitogen-activated protein kinase 3; MKK6 = mitogen-activated protein kinase 6; mTOR = mechanistic target of rapamycin; NF-κB = nuclear factor κB; Nrf2 = nuclear factor erythroid 2–related factor 2; PFK2 = 6-phosphofructo-2-kinase; PINK-1 = PTEN-induced putative kinase-1; PI3K = phosphatidylinositol-3-kinase; SDF-1 = stromal cell–derived factor-1; SHP = small heterodimer partner; Smac = second mitochondria–derived activator of caspases; TLR4 = toll-like receptor 4; TRPV1 = transient receptor potential vanilloid 1; VEGF = vascular endothelial growth factor; VEGFR2 = vascular endothelial growth factor receptor 2.

## Data Availability

Data sharing is not applicable to this article as no datasets were generated or analyzed during the current study.
